# 60 Sustainable impact of an outdoor exercise program on health outcomes of older adults: a quasi-randomized controlled trial with a 3-month follow-up

**DOI:** 10.1093/eurpub/ckae114.110

**Published:** 2024-09-26

**Authors:** Katharina Zwingmann, Torsten Schlesinger, Katrin Müller

**Affiliations:** Chemnitz University Of Technology, Institute of Human Movement Science and Health, Chemnitz, Germany

## Abstract

Older people benefit in several health-promoting outcomes from needs-oriented exercise programs in their immediate living environment. For a low-threshold, and thus sustainable and cost-effective design of exercise programs a transfer to self-organisation is favourable. The aim of our study is to examine the health-promoting impacts of an outdoor exercise program developed for older persons, as well as its sustainability after 3 months.

Ninety-five persons aged 60+ were randomized according to their residence (IG: n = 40, mean age = 72.35 years, 24 female; KG: n = 55, mean age = 75.05 years, 34 female). An age-adjusted exercise program was carried out over 12 weeks (2x/week á 45-60min; adherence: 90.9%) by professional exercise instructors in urban parks. During this instructor-led exercise phase, seven participants were trained as mentors, and thus, enabled to continue the exercise program for further 12 weeks (1-2x/week á 45-60min; adherence: 2-3x/month = 11.5%, 1-2x/week = 88.5%). Before (T1) and after the instructor-led exercise phase (T2) as well as after the mentor-led exercise phase (T3), health outcomes (e.g., walking distance/6MWT, neurocognitive performance/DSST) of IG and CG (no intervention) were assessed and analysed using paired t-Tests.

75.1% of IG participants completed both phases of the exercise program. Neurocognitive performance improved significantly for IG between T1 and T2 (+2.32 correct number-symbol matches; p = .003; d = -.509) and remained stable between T2 and T3 (+0.15 correct matches; p = .863; d = -.028) while for CG the opposite development was observed (T1-T2: -0.6 correct matches; p = .451; d = .102; T2-T3: +2.18 correct matches; p = .004; d = -.423). IG improved their walking distance for T1-T2 by 21.39m (p = .172; d = -.223), which could be maintained for T2-T3 (+5,1m; p = .648; d = -.074). Improvements in CG for walking distance were smaller (T1-T2: +2.8m; p = .690; d = -.055; T2-T3: +14.26m, p = .315; d = -.147).

The mentoring of the exercise groups proved to be very successful, as evidenced by the maintained effects in the mentor-led phase, ensuring sustainability of health-promoting interventions.

(Funded by the Free State of Saxony, “Demographics” #100535625)

